# The Effects of Non-Nutritive Artificial Sweeteners, Aspartame and Sucralose, on the Gut Microbiome in Healthy Adults: Secondary Outcomes of a Randomized Double-Blinded Crossover Clinical Trial

**DOI:** 10.3390/nu12113408

**Published:** 2020-11-06

**Authors:** Samar Y. Ahmad, James Friel, Dylan Mackay

**Affiliations:** 1 Richardson Centre for Functional Foods and Nutraceuticals, Department of Human Nutritional Sciences, University of Manitoba, 196 Innovation Drive, Winnipeg, MB R3T 2E1, Canada; James.Friel@umanitoba.ca (J.F.); Dylan.Mackay@umanitoba.ca (D.M.); 2 Department of Community Health Sciences, Rady Faculty of Health Sciences Winnipeg, University of Manitoba, MB R3T 6C5, Canada

**Keywords:** non-nutritive sweetener, aspartame, sucralose, gut microbiome, randomized clinical trial, protocol

## Abstract

Non-nutritive artificial sweeteners (NNSs) may have the ability to change the gut microbiota, which could potentially alter glucose metabolism. This study aimed to determine the effect of sucralose and aspartame consumption on gut microbiota composition using realistic doses of NNSs. Seventeen healthy participants between the ages of 18 and 45 years who had a body mass index (BMI) of 20–25 were selected. They undertook two 14-day treatment periods separated by a four-week washout period. The sweeteners consumed by each participant consisted of a standardized dose of 14% (0.425 g) of the acceptable daily intake (ADI) for aspartame and 20% (0.136 g) of the ADI for sucralose. Faecal samples collected before and after treatments were analysed for microbiome and short-chain fatty acids (SCFAs). There were no differences in the median relative proportions of the most abundant bacterial taxa (family and genus) before and after treatments with both NNSs. The microbiota community structure also did not show any obvious differences. There were no differences in faecal SCFAs following the consumption of the NNSs. These findings suggest that daily repeated consumption of pure aspartame or sucralose in doses reflective of typical high consumption have minimal effect on gut microbiota composition or SCFA production.

## 1. Introduction

Non-nutritive sweeteners (NNSs) are chemicals that produce an intense sweet taste at a very low concentration compared to that of caloric sweeteners such as sucrose, dextrose and high-fructose corn syrup. Additionally, known as non-caloric or artificial sweeteners, NNSs have grown increasingly popular since their introduction to the food and beverage market. This is a result of their low cost, their low- or zero-calorie counts, and their noted health benefits for weight loss/management and the normalization of blood glucose levels [1,2].

In Canada, the approved NNSs are acesulfame potassium, neotame, sucralose, aspartame, monk fruit extract, steviol glycosides and erythritol [3]. The NNSs sucralose and aspartame were evaluated in this study because they are used more often than others, especially in diet soft drinks [4,5,6].

Health Canada has defined the acceptable daily intake (ADI) of sucralose as 9 mg/kg body weight and 40 mg/kg body weight for aspartame [7]. A 150-pound individual would need to consume approximately twenty 355 mL servings of diet coke containing 131 mg aspartame or fourteen 341 mL servings of diet ice teas containing 41 mg sucralose to reach the respective ADIs mentioned above.

The human gut microbiota hosts up to 100 trillion bacteria in the large intestine alone [8], where the largest colonization of the diverse human microbiota is present. Microbial communities are unique to every host, and they can respond instantly to any changes in the host diet or can be affected by different environmental factors [9]. Moreover, many changes in the diet can affect short-chain fatty acids (SCFAs) [10]. SCFAs are the main end-products resulting from the fermentation of non-digestible carbohydrates that become accessible to the gut microbiota [11]. SCFAs have a role in glucose metabolism, lipid metabolism, appetite regulation, and the immune system [12]. Bacteroidetes and Firmicutes are the two bacterial phyla that dominate almost 90% of the adult gut microbiota, while a minority of the bacteria are represented by Proteobacteria, Verrucomicrobia, Fusobacteria and Cyanobacteria [13].

The human microbial ecosystem has more than a thousand microbial species that play different roles, including maintenance of host immunity, metabolism, endocrine signalling [14], drug metabolism, vitamin production, and carbohydrate metabolism, in addition to the breakdown of indigestible polysaccharides into short-chain fatty acids, such as acetate, butyrate, formate and propionate [15,16]. Any change in the number, composition or quality of the gut microbiome may affect the different physiological roles of these microbes and cause gut microbiota dysbiosis [17]. Unhealthy diets that are rich in saturated fat and refined sugar, along with decreased physical activity, have been linked to gut microbiota dysbiosis, which may cause impaired glycaemic control [18].

In recent years, research interest has been focused on the interaction between the microbiota and the host and how the human gut microbiota composition may potentially have an effect on the development of certain diseases, such as metabolic syndrome, obesity, type 2 diabetes and type 1 diabetes [19].

More recently, NNS research has focused on the effect of non-nutritive artificial sweeteners on the gut microbiota due to their possible impact on insulin resistance, obesity, and inflammation [20]. Saccharin consumption in mouse models has been shown to induce marked glucose intolerance via gut microbiota dysbiosis [21]. Some microbes in the gut microbiota may have the ability to metabolize NNSs, which can cause a shift in the normal bacterial balance [6]. It is also possible that NNS intake may have a bacteriostatic effect on certain gut microbes, causing changes to the microbiome composition [22,23]. However, it is important to highlight the paucity of human studies on the gut microbiome in relation to NNS consumption [21]; more research is needed in this area, taking into consideration many of the limitations that are present in the existing NNS randomized clinical trial [24].

Suez et al. (2014) reported that four out of seven healthy subjects who consumed the United States Food and Drug Administration (FDA’s) maximal ADI of saccharin during a 5-day period presented with a poorer glycaemic response after this intervention than before. Moreover, there was a pronounced change in the microbiome composition of the four participants with a poor glycaemic response compared to that in other participants [21].

To the best of our knowledge, there is no clinical trial that has investigated the effects of aspartame or sucralose, the most commonly used sweeteners in Canada, at levels reflecting the high habitual diet soda intake in healthy participants, or has addressed their possible effect on the gut microbiome. Therefore, this study aimed to determine the effect of sucralose and aspartame consumption on gut microbiota composition, diversity, and community structure and the effect of sucralose and aspartame consumption on SCFAs (microbiota metabolites); this is the secondary outcome of the previously published clinical trial [25].

## 2. Materials and Methods

### 2.1. Study Design

This study is a randomized, double-blind crossover and controlled clinical trial that took place in the Richardson Centre for Functional Foods and Nutraceuticals (RCFFN) at the University of Manitoba in Winnipeg, Canada. Primary outcome results of this study have been reported elsewhere [25]. The study protocol was reviewed and approved by the University of Manitoba Bannatyne Campus Biomedical Research Ethics Board (BREB) in Winnipeg, Manitoba, Canada. This trial was registered at ClinicalTrials.gov (Identifier: NCT02569762) in October 2015.

Participants gave consent to follow a 12-week diet regimen in a crossover design. For the first four weeks, all participants went through a baseline period, where no artificial sweeteners were consumed. During weeks 5 and 6, nine of the participants consumed aspartame, and eight of the participants consumed sucralose. For weeks 7 through 10, all participants underwent a washout period, where no artificial sweeteners were consumed. Last, during weeks 11 and 12, all participants consumed the sweetener that they did not previously consume. The study schedule of enrolment, interventions, and assessments is summarized in Table 1.

### 2.2. Study Participants

Adult males and females aged 18–45 years were selected for inclusion in this study from the Winnipeg Region. Posters and flyers were posted and distributed in Winnipeg. An advertisement was placed in the local newspapers and via the internet (i.e., https://www.kijiji.ca) to notify people of the study. The preliminary eligibility of individuals who expressed an interest was first determined via short telephone questionnaire. Then, participants came to the research center for screening to determine full eligibility. Participants were remunerated for participation in the study by giving each participant who completed the study a USD 200 gift card.

A total of 17 participants completed the study (10 females and seven males); eight participants were allocated to the first treatment group, two participants dropped out from the first group without providing a reason and did not receive the allocated intervention; nine participants were allocated to the second group. The baseline characteristics of the study participants who completed the study are shown in Table 2. All participants were young healthy adults with a mean age of 24 ± 6.8 years, a normal body mass index (BMI) (22.9 ± 2.5 kg/m^2^) and biochemical markers within the normal range. Figure S1 is the flow diagram of the trial according to Consolidated Standards of Reporting Trials (CONSORT) guidelines [25].

### 2.3. Inclusion and Exclusion Criteria

Participants who were healthy and non-diabetic were included in the study. Inclusion criteria were 18–45 years old of age, body mass index (BMI) of 20–25 (i.e., normal weight), fasting blood glucose ˂ 5.7 mmol/L (i.e., normal fasting glucose), and people who do not regularly consume NNS (non-consumers). Women with regular cycles who were not taking oral contraceptive pills were included in the study, and they all began the study at approximately the same phase (follicular) of their respective menstrual cycles.

Exclusion criteria included a history of alcohol or drug abuse, use of antibiotic medications or probiotics within the 6-month period prior to the study, and any acute or chronic medical conditions that could potentially affect outcomes. Such medical conditions included metabolic or gastrointestinal disorders (e.g., diabetes, malabsorption syndrome, inflammatory bowel disease, irritable bowel syndrome, celiac disease, phenylketonuria); medications that impact glucose metabolism or the gut microbiome (e.g., metformin); changes in gastric pH (e.g., proton pump inhibitors) or gastric emptying (e.g., metoclopramide); or known allergies, sensitivities or contraindications to aspartame or sucralose. Pregnant and lactating women, and those planning to become pregnant, were excluded because there is not enough evidence about the negative health effect of NNSs during pregnancy or lactation. Investigators could decide to remove participants from the study if the participants decided to stop drinking the artificially sweetened beverage as described by the protocol or if they began using any steroids or beta agonists (orally, intranasal or inhaled) within a week of any oral glucose tolerance test.

### 2.4. Randomization

Eligible participants underwent baseline assessments and were randomly assigned by trial coordinator to two groups, an aspartame-then-sucralose group or a sucralose-then-aspartame group, after enrolment in the trial. The randomization codes were concealed in opaque sealed envelopes and were released to a staff member after all baseline measurements were completed. The order of the sequences in the envelopes was created by coin-flip. The assignment of this intervention was blinded for both the investigators and the participants. For blinding purposes, the beverages were prepared by a kitchen staff and later distributed in identical bottles labelled “A” or “B” by aperson outside the research team.

### 2.5. Dietary Recommendation during the Time of the Study

Participants were advised to avoid consuming any NNSs during the study period and were taught about the hidden sources of any NNSs in different foods, beverage products and medication. Examples of other NNSs that were avoided are aspartame, acesulfame potassium, neotame or E961, saccharin, sucralose, stevia and monk fruit extract. Additionally, the participants received recommendations regarding their caffeinated beverage intake because caffeine ingestion has an effect on glucose uptake rates [26]; such intake was limited to two cups (250 mL) per day of drinks such as tea, coffee, energy drinks and soft drinks. In addition, the participants limited their alcohol intake to no more than two units of alcohol per day (unit = 10 mL pure alcohol) due to the effect of alcohol on blood glucose and insulin levels and the gut microbiome [27,28]. During the time of the study, participants refrained from consuming any probiotic supplements or foods containing probiotics, such as kefir, coconut kefir, yogurt, natto, miso soup, raw cheese, kombucha tea, tempeh, fermented soybean and fermented cabbage. Participants were restricted from using ibuprofen (Advil and Motrin) during the study; only acetaminophen (Tylenol) was allowed to be taken if needed, and the study coordinator had to be notified regarding the use of any other medications. Probiotics and ibuprofen have an effect on gut microbiota function and composition [29,30].

### 2.6. Interventions

Some participants received aspartame during weeks 5 and 6, while the others participants consumed sucralose. During weeks 11 and 12, all participants consumed the sweetener that they did not previously consume. The amount each participant consumed was determined based on the average body weight in adults to meet 14% of the ADI for aspartame and 20% of the ADI for sucralose. These dosages are based on the patterns of regular soft drink intake in Canadian men and women [5]. This dosage level is high but reasonable and realistic, reflecting the intake of consumers, who drink approximately three cans of diet soda a day. Fourteen percent of the ADI for aspartame is approximately equivalent to 0.425 g of aspartame (10 packets of aspartame), while 20% of the ADI for sucralose is approximately equivalent to 0.136 g of sucralose (approximately 10.5 packets of sucralose). Participants were given the sweeteners in a blinded fashion with beverages in identical bottles labelled “A” or “B”.

Beverages were given in two 500-mL containers. The Aspartame beverage: 1000 mL of water, 0.08 g of citric acid, 0.037 g of pure lemon extract (Club House brand, McCormick London On, Canada), and 0.425 g of pure aspartame powder (HerbStoreUSA, Walnut, CA, USA). Aspartame was dissolved in the water using a high-frequency ultrasonic bath for 10 min. The sucralose beverage consisted of 1000 mL of water, 0.08 g of citric acid, 0.037 g of pure lemon extract (Club House brand, McCormick London, ON, Canada), and 0.136 g of pure sucralose powder (HerbStoreUSA, Walnut, CA, USA). During the washout period, participants were asked to maintain their habitual water and food intake.

### 2.7. Assessment and Evaluation

The beverage was distributed in two 500-mL bottles, and the participants were instructed to return all empty bottles for counting purposes.

A visual analogue scale (VAS, fixed length 100 mm) was used to measure physical comfort, motivation to eat, palatability, energy and fatigue in participants during the intervention period [31]. The VAS score allowed us to look at changes within individuals receiving each beverage [25].

### 2.8. Study Measures

All anthropometric and biochemical measurements were performed on the first day after a 10- to 12-h overnight fast. Fasting blood draw performed by a registered nurse (RN) at time 0. Participants were given a 75 g glucose tolerance test beverage (Trutol) followed by additional blood samples taken at 15, 30, 45, 60, 90 and 120 min. During the intervention periods, a baseline sample was drawn at day 1 and then at the beginning and end of each treatment period, i.e., days 28, 42 and 84. For female participants, fasted blood draws were scheduled after the end of the monthly menstrual cycle. Detailed measurements were prescribed elsewhere [25].

### 2.9. Sample Collection

Blood and faecal samples were collected. Faecal samples were collected from participants on days 1, 28, 42 and 84. All participants were instructed to provide two faecal samples. Sterile faecal sample collection tubes were given to each participant. They were advised to fill each collection tube with approximately a third of the stool sample each from 3 different random sites of the stool. They were instructed to keep the sample in cold storage (~4 °C) immediately after collection. The frozen stool samples were delivered to our center using safe transportation packages with ice (provided by us). Once the samples were collected at the center, the samples were stored at −80 °C for later microbiota analysis. The target period from the time from sampling to delivery was to be as short as possible, no more than 6–8 h. The frozen stool samples were shipped in a Styrofoam container packed with dry ice to handling laboratory (microbiome insights) in Vancouver (BC, Canada) for analysis [32].

### 2.10. Faecal Microbiota

Frozen stool samples were processed for bacterial DNA extraction using the MagAttract^®^ PowerSoil^®^ DNA KF Kit (QIAGEN Inc., Toronto, ON, Canada) in combination with KingFisher^®^ Flex (ThermoFisher Scientific, Waltham, MA, USA) following MoBio’s instructions on a KingFisher robot, which included a bead-beating step to aid mechanical lysis of microbial cell walls. The DNA concentration was quantified using a NanoDrop 2000 spectrophotometer (Thermo Scientific, Waltham, MA, USA). Bacterial 16S rRNA genes were polymerase chain reaction (PCR)-amplified using dual-barcoded primers targeting the V4 region (515F 5′-GTGCCAGCMGCCGCGGTAA−3′, and 806R 5′-GGACTACHVGGGTWTCTAAT−3′) according to the protocol of Kozich et al. (2013) [33].

The Illumina MiSeq platform was used to perform the 300-bp paired-end sequencing reaction using the MiSeq reagent kit version 3 (300-cycle; Illumina, San Diego, CA, USA). The 16Sv4 amplicons produced from human faecal samples were sequenced on a MiSeq platform. Sequences were denoised and taxonomically classified using Greengenes version 13_8 as the reference database and assigned into 97% similarity operational taxonomic units (OTUs) with the mothur software package (v.1.39.5) [33] following the recommended procedure on the website [34]. The resulting database had 55,886 OTUs. On average, 28,802 quality-filtered reads were generated per sample. Sequencing was performed in microbiome insights laboratory in Vancouver (BC, Canada). Beta diversity across samples was estimated by exclusion of OTUs occurring with a count of less than 3 in at least 10% of the samples and then computed Bray–Curtis indices. Principal coordinate analysis (PCoA) ordination was used to visualize beta diversity and emphasize differences across samples. The DESeq2 package was used to determine different abundant taxa among different variables such as the type of treatment received, the sequence of the treatment received before the washout period and the period whether it is before or after the washout period. Permutational analysis of variance (*adonis* R function, or Permanova) determined Permutational Analysis of Variance (PERMANOVA) tests for significant variations in the whole microbiome between continuous variables. The samples were randomly re-allocated to the different sample categories (Monte-Carlo Permutations), and the between-category variations were compared to the true between-category differences. PERMANOVA used the sample-to-sample distance matrix (Bray-Curtis) directly to carry out the calculation.

### 2.11. Faecal SCFA Analysis

SCFA extraction was performed as described previously in the literature [35]. Fecal suspension was performed in Milli-Q-grade H_2_O and homogenized using MP Bio Fast Prep for 1 min at 4.0 m/s. Acidification of faecal suspensions (pH of 2.0) was performed by adding 5 M HCl. After incubation of the acidified faecal suspensions, they were centrifuged at 10,000 revolutions per minute (RPM) to separate the supernatant. A final concentration of 1 mM was reached for the faecal supernatants. Then, the SCFA extracted supernatants were stored in 2 mL GC vials. Detection of SCFAs was performed through gas chromatography (Thermo Trace 1310) coupled to a flame ionization detector (Thermo). The column used in the SCFA detection is ‘Thermo TG-WAXMS A GC Column, 30 m, 0.32 mm, 0.25 um’, which is very similar to the methods used in the literature [35].

### 2.12. Statistical Analysis

A minimum sample size of 12 was required for the glucose metabolism outcome. The number of faecal samples analysed was 64 for 17 participants, four samples per participant, two baselines and two endpoints. There were four missing samples at different timepoints.

**Figure 1:**
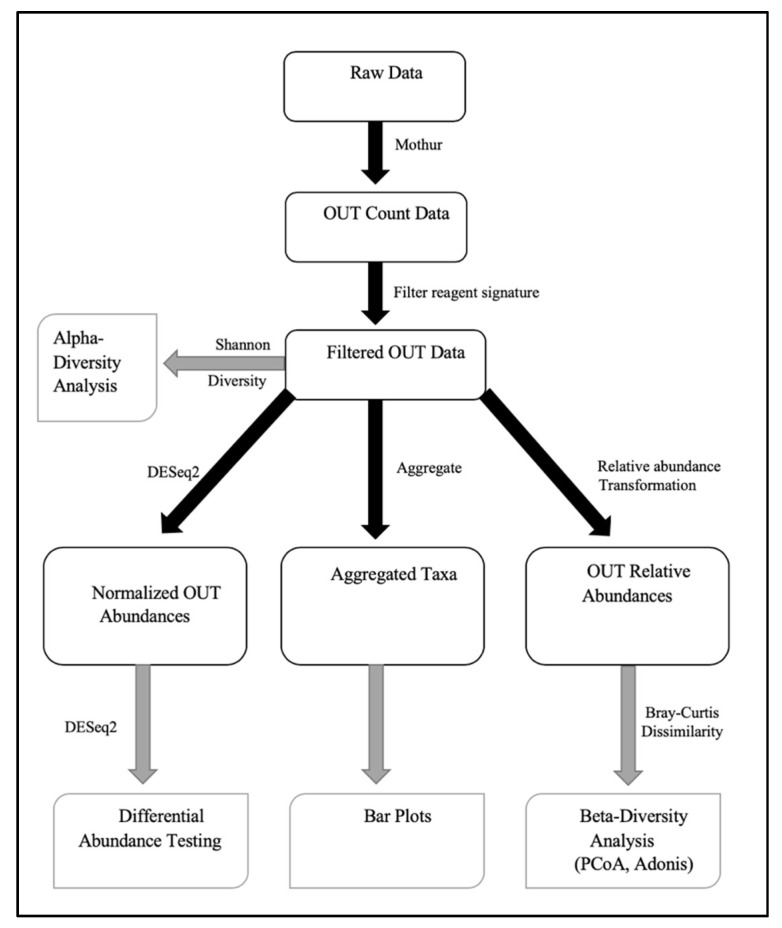
Analytical flow chart.

A linear mixed-effects model was used to test the significance of diversity differences. Variation in the community structure was assessed by permutational multivariate analyses of variance (PERMANOVA) with the treatment group being the main fixed factor and using 9999 permutations for significance testing [36]. The Adonis test was used to test for statistically significant differences in beta diversity among sequence factor(s). The R environment was used to conduct all analyses. The analytical flowchart is shown in Figure 1. A linear mixed-effects model was used to test statistical significance among different short-chain fatty acids. For all analyses, *p* values < 0.05 were considered significant.

### 2.13. Faecal Microbiota Analysis

**Figure 2:**
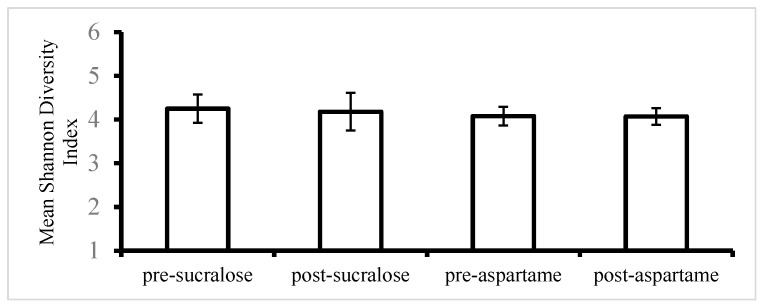
Change in the mean Shannon diversity index after sucralose or aspartame treatment (*n* = 17). Between-group comparison by the linear mixed effect model. Values are expressed as the means ± standard error of mean (SEM).

The faecal microbiota was characterized in 17 healthy adults before and after aspartame or sucralose drinks. Alpha diversity estimation was performed with the Shannon index on raw operational taxonomic unit (OTU) abundance tables after removing the contaminants [37]. The gut microbiota richness and evenness (Shannon index) in the faecal samples did not change following the introduction of aspartame or sucralose treatments for 14 days (Figure 2). The number of observed species was 245. A linear mixed-effects model was used to test for differences in the Shannon diversity index, and there were no differences seen before or after treatments with sucralose or aspartame (Table 3).

## 3. Results

### 3.1. Participants

The primary outcome was to assess the effect of aspartame and sucralose on glucose metabolism in healthy adults and it was shown that daily consumption of pure aspartame or sucralose for 14 days did not measurably influence glucose metabolism or insulin sensitivity in healthy adults [25]. Based on the returned empty-drink-bottle counts, there were no differences in treatment consumption. The mean bottle count adherence for aspartame treatment was 99.47 ± 1.49% compared to 100% for the sucralose treatment.

**Figure 3:**
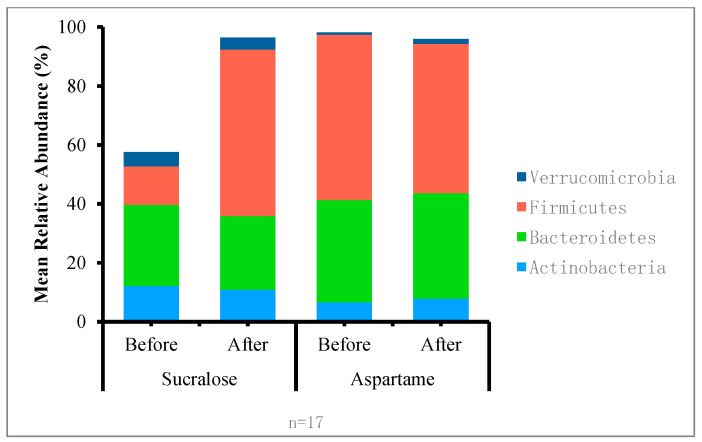
Mean relative abundance of the four dominant microbiota phyla of the human gut by treatment group before and after the administration of aspartame or sucralose drinks.

**Figure 4:**
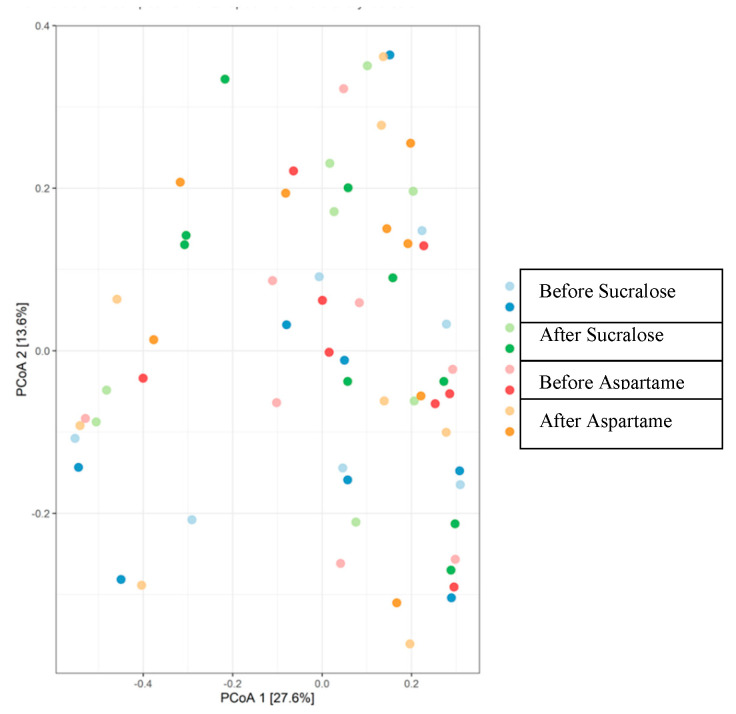
Principal coordinates analysis of the microbiota community structure (unweighted UniFrac distance) before and after treatment with sucralose or aspartame; statistical comparison by permutational multivariate analysis of variance (PERMANOVA) with 500 permutations. (*n* = 17).

Across treatment groups, the relative proportions of the most abundant bacterial phyla and genus-level taxa were similar before and after treatments (*p* > 0.05) (Table 4 and Figure 3), and the microbiota community structure did not show any obvious differences (Figure 4, PERMANOVA *p* = 0.99). The graphical representation of the principal coordinates analysis of the microbiota community structure after treatment with sucralose or aspartame is shown in Figure 4. The tests are based on Bray–Curtis dissimilarity distances and 500 permutations.

The permutational analysis of variance (Adonis R function) showed a difference in beta diversity between the sequence factor, which could suggest that the order of NNS received participants received influenced beta diversity (*p* < 0.05). There were no differences among other factors such as the type of treatment received or the period when they received the treatment (*p* > 0.05) (Table 5).

A linear model was used for the test, and the reduced terms of the likelihood ratio test was ~1. Two OTUs were identified during the differential abundance testing (DESeq2 and R package). The two bacterial phyla that were most differentially abundant among treatments were Bacteroidetes (genus Bacteroides, unclassified) (Padj < 0.05) and Firmicutes (order Clostridiales, unclassified) (Padj < 0.05).

### 3.2. Faecal Metabolomic Analysis

**Figure 5:**
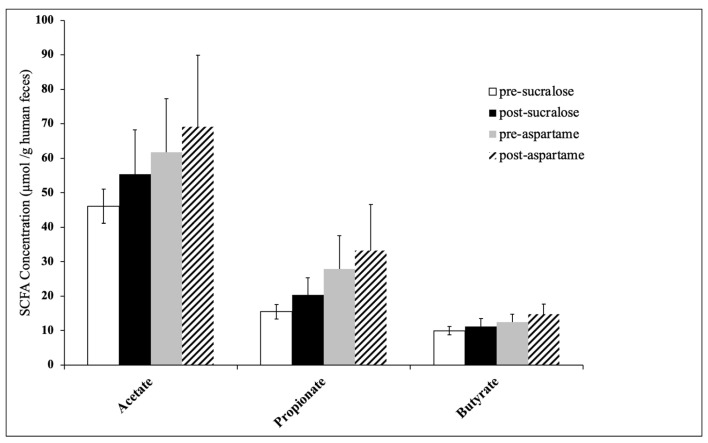
Short-chain fatty acid concentration from the human faeces metabolomics analysis before and after sucralose or aspartame treatments. Data are presented as the means ± standard error (SE), *n* = 14–16.

Changes in the SCFA concentration from metabolomics analysis in healthy participants are shown in Table 6. Concentrations were normalized to the amount of input material (mmol SCFA/kg human faeces). Sucralose or aspartame treatments did not cause changes in six faecal metabolites, including acetate, propionate, butyrate, isovaleric acid, valeric acid and hexanoic acid. A linear mixed effects model was used to test statistical significance among different variables. Figure 5 shows the predominant SCFA acetate, butyrate and propionate of bacterial origin.

## 4. Discussion

This is one of the first studies to assess repeated daily oral intake of beverages sweetened with a pure powder of sucralose or aspartame by healthy adults in a double-blinded, randomized, crossover study. The main outcome of this clinical trial was to determine the effect of short-term consumption of aspartame or sucralose on glucose metabolism [25]. The results reported here were the secondary outcome of this clinical trial, and were used to measure the effect of repeated daily intake of NNSs on the gut microbiota and SCFAs of bacterial origins.

We found that daily oral consumption of beverages sweetened with 136 mg/day sucralose or 425 mg/day aspartame did not measurably affect the gut microbiota in healthy participants. Additionally, we did not detect a change in the gut microbiota structure (PERMANOVA *p* = 0.99). SCFAs were also unaffected by aspartame and sucralose consumption. The doses of NNS used in this study resemble an intake of approximately three 355 mL cans of beverages per day [4].

Far fewer human trials than animal studies have assessed the effect of non-nutritive sweeteners on gut microbiota. For example, Suez et al. [21] showed that four out of seven healthy adults developed glucose intolerance after daily intake (for 5 days) of a high dose of saccharine (> 5 mg/kg/day). Moreover, it was shown that gut microbiota dysbiosis was associated with changes in glucose metabolism, and this was demonstrated when the researchers transferred the faecal microbiota from the participants into a germ-free mouse. Glucose intolerance was also observed in recipient mice [21]. However, due to the doses used, the different chemical structures, and metabolic fates of different types of NNSs, we cannot extrapolate the results of Suez et al. to other types of sweeteners, such as aspartame or sucralose. In particular, the dose of saccharine used in that trial exceeded the FDA’s ADI, which might have an impacted the outcomes measured in that study [38].

In our clinical trial, the relative abundance of the dominant microbiota phyla did not change after 14 days of ingestion of sucralose or aspartame drinks, which suggests that sucralose or aspartame was not causing dramatic changes in gut microbiota richness or evenness. This is consistent with Frankenfeld et al., (2015) who found no effect of aspartame or acesulfame-k consumption on bacterial abundance in 31 adults [39].

Additionally, there was no change in the gut microbiota structure, which supports previous metabolic studies in animals and humans. Many of the acute studies of the biological fate of sucralose in animals and humans have shown that sucralose is not absorbed but is eliminated unchanged in faeces, which makes it unlikely to be a substrate for gut microbiota [40,41,42,43], which support sucralose safety.

Unlike our findings, pervious animal studies have shown different effects of NNS on different bacterial genera [22,44,45,46]. It has been suggested that these disruptions in the gut microbiome might interfere with host gut functions and could impact health [47]. Additionally, some animal studies have shown changes in of SCFAs [22,23,45,46].

Extrapolating NNS animal data to humans must be done cautiously as animal data is often a poor predictor human response. There is also a paucity of clinical trials measuring the effect of aspartame or sucralose on the gut microbiota. 

The strength of our study is the design, which was double-blind, and randomized, with a crossover, and we included male and female participants. We believe that another strength of our study comes from the use of the pure form of sucralose and aspartame powders in order to steer clear of other ingredients that are present in diet soda or packaged NNSs (i.e., Splenda) and then to examine the overall effect of diet soda.

Some limitations should be noted. For example, the 14-day intervention period might not be enough to observe changes in the gut microbiota or the SCFA, but this timeframe happened to accommodate the washout periods and multiple treatment periods in this crossover trial and minimize the drop-out rate that might result from a prolonged duration of study. There could also be a limitation in the study design as this study was powered mainly to look at the effect of NNS on blood glucose levels, and not powered to look at the effect of NNS on the gut microbiota, which was an exploratory outcome.

Aspartame and sucralose are the most common NNSs used in Canada [5]. Since the extensive introduction of artificial sweeteners to our diet, their consumption has been associated with an increased risk of overweight, obesity and diabetes in some populations [48]. However, the lack of randomized clinical trials in healthy individuals involving NNS and gut microbiome studies makes this investigation an important addition to the literature and informs further research on the relationships between NNSs, glucose metabolism and the gut microbiota in the context of human health and disease.

## 5. Conclusions

In conclusion, our study showed that aspartame and sucralose did not cause measurable changes in the gut microbiota or SCFAs after 14 days of a realistic daily intake in healthy participants. This is in contrast to the many of microbiome studies conducted in animal models, however, their applicability to human health and disease may be limited.

## Figures and Tables

**Table 1:** Summary of the study schedule for enrolment, intervention and assessment.

Assessments	Screening & Enrollment	Visit 1	Visit 2	Visit 3	Visit 4
Day in study		1	28	42	84
Week in study	−2	1–4	5,6	7–10	11,12
General information form	✓				
Informed consent	✓	✓			
Medical history	✓	✓			
Weight	✓	✓	✓	✓	✓
Non-nutritive sweetener supplementation ^1^			✓		✓
Blood draw ^2^ (1.5 mL) session		✓	✓	✓	✓
Stool sample collection		✓	✓	✓	✓
Laboratory measurements in plasma: glucose, insulin, glucagon, GLP-1, leptin		✓	✓	✓	✓
Laboratory measurements in stool: fecal microbiome, fecal short-chain fatty acids (SCFA)		✓	✓	✓	✓
Oral glucose tolerance test (OGTT) ^3^		✓	✓		✓
Fasting blood glucose (FBG) test ^4^	✓	✓	✓	✓	✓
Visual analogue scales (taste panel) ^5^		✓	✓	✓	✓
Diet history questionnaire	✓				
Food diary ^6^		✓	✓	✓	✓
Adverse event log		✓	✓	✓	✓

^1^ This will be either aspartame or sucralose; visits to collect the supply will vary. ^2^ There will be five blood draws after the visits, and they will be conducted at the Richardson Centre for Functional Foods and Nutraceuticals (RCFFN). ^3^ OGTT will involve drinking a sweet liquid containing 75 g of glucose. ^4^ FBG will be conducted after a 10–12 h fast. ^5^ This test is to measure the participants’ tolerance to the sweetener mixed into beverages. ^6^ Food diaries documenting 2 weekdays and 1 weekend day.

**Table 2:** Characteristics of participants at baseline ^1^.

Variables	Value
Total participants (F/M)	17 (10/7)
Age (years)	24 ± 1.64
Body weight (kg)	68.9 ± 2.54
BMI (kg/m2)	22.9 ± 0.6
FBG (mmol/L)	5.3 ± 0.1
Caffeine (mg) (*n* = 16) *	176.87 ± 46.68
Protein (g) (*n* = 16) *	70.87 ± 7.33
Carbohydrate (g/day) (n = 16) *	228.60 ± 24.57
Fiber (g) (*n* = 16) *	17.43 ± 2.30
Total fat (g) (*n* = 16) *	72.04 ± 7.78
Aspartame (mg) (*n* = 15) *	11.81 ± 2.99
Sucralose (mg) (*n* = 12) *	65.83 ± 48.65
Total SCFA (mmol/kg)	78.57 ± 11.18
Acetic acid (mmol/kg)	46.87 ± 24.94
Propionic acid (mmol/kg)	16.61 ±10.57
Butyric acid (mmol/kg)	10.51 ± 6.26
Isovaleric acid (mmol/kg)	2.27 ± 1.73
Valeric acid (mmol/kg)	1.86 ± 1.87
Hexanoic acid (mmol/kg)	0.42 ± 0.74

^1^ Values are expressed as the means ± SEMs unless otherwise indicated; * these intakes reflect the participants’ food intake over the 12 months prior to enrolment in the clinical trial. Concentrations were determined from plasma; F, females; M, males; BMI, body mass index; short chain fatty acid, SCFA; FBG, fasting blood glucose.

**Table 3:** Shannon diversity index, which shows the microbiota richness and evenness by treatment group (*n* = 17).

	Sucralose				Aspartame			
	Pre-Treatment	Post-Treatment	% Change	*p*-Value *	Pre- Treatment	Post-Treatment	% Change	*p*-Value *
**Shannon index** (**alpha diversity**)	4.25 ± 0.79	4.18 ± 0.81	−1.64	0.63	4.08 ± 0.82	4.07 ± 0.65	−0.24	0.96

* Comparison by Linear Mixed Effects Model between treatment groups. Values are expressed as the means ± standard deviations (SDs).

**Table 4:** Median relative abundance of the 5 most abundant genus-level taxa within the 4 most dominant * phyla before and after treatment with sucralose or aspartame drinks for 14 days in healthy adults (*n* = 17).

**	Phylum Genus	Pre-Treatment		Post-Treatment		*p*-Value	
		Sucralose	Aspartame	Sucralose	Aspartame	Sucralose	Aspartame
	**Actinobacteria**	0.121	0.103	0.043	0.031	0.64	0.96
	f__Clostridiaceae_ unclassified	0.000	0.000	0.000	0.000	0.55	0.31
	g__Bifidobacterium	0.102	0.095	0.026	0.028	0.54	0.88
	g__Collinsella	0.010	0.009	0.008	0.005	0.96	0.72
	g__Eggerthella	0.000	0.000	0.000	0.000	0.80	0.31
	g__Slackia	0.000	0.000	0.000	0.000	0.78	0.89
	**Bacteroidetes**	0.131	0.215	0.374	0.409	0.61	0.92
	g__[Prevotella]	0.000	0.000	0.000	0.000	0.21	0.48
	g__Alistipes	0.003	0.009	0.013	0.013	0.23	0.68
	g__Bacteroides	0.035	0.053	0.075	0.098	0.29	0.96
	g__Parabacteroides	0.001	0.004	0.005	0.005	0.26	0.16
	g__Prevotella	0.028	0.019	0.101	0.014	0.35	0.47
	**Firmicutes**	0.517	0.548	0.533	0.530	0.18	0.54
	F_Ruminococcaceae_ Unclassified	0.036	0.028	0.027	0.027	0.30	0.15
	g__Blautia	0.075	0.077	0.099	0.087	0.88	0.64
	g__Coprococcus	0.036	0.043	0.033	0.024	0.96	0.76
	g__Faecalibacterium	0.026	0.024	0.066	0.033	0.41	0.10
	G_Roseburia	0.040	0.018	0.024	0.021	0.17	0.43
	Verrucomicrobia	0.000	0.000	0.000	0.000	0.92	0.44
	g__Akkermansia	0.000	0.000	0.000	0.000	0.92	0.44

* Dominant taxa are taxa with >0% median relative abundance. Comparisons pre- and post-treatments were performed by the Wilcoxon rank sum test. ** The different colors in the first column highlights the 5 most abundant genus-level taxa within the 4 most dominant phyla.

**Table 5:** PERMANOVA ^1^ analysis of different factors with the unweighted UniFrac distance matrix.

Adonis Model ^1^	*R* ^2^	*p*-Value
Treatment	0.012	0.99
Sequence	0.03	0.02 *
Period	0.01	0.78
Residuals	0.94	NA
Total	1.00	NA

^1^ The Adonis test uses permutational multivariate analysis of variance (PERMANOVA) to test for statistically significant differences in beta diversity among sequence factor(s). All analyses were conducted in the R environment. * A *p*-value of < 0.05 was considered significant.

**Table 6:** Short chain fatty acid (SCFA) concentration (mmol SCFA/kg human faeces) from the metabolomics analysis in healthy participants.

SCFA Concentration (µmol SCFA/g Human Faeces)	Sucralose Treatment			Aspartame Treatment		
	Pre-Sucralose	Post-Sucralose	*p*-Value *	Pre-Aspartame	Post-Aspartame	*p*-Value *
Acetate	46.13 ± 4.97	55.37 ± 12.92	0.49	61.76 ± 15.47	69.08 ± 20.84	0.79
Propionate	15.48 ± 2.11	20.35 ± 5.04	0.39	27.83 ± 9.69	33.24 ± 13.37	0.75
Butyrate	9.95 ± 1.21	11.17 ± 2.32	0.60	12.47 ± 2.34	14.78 ± 2.87	0.56
Isovaleric acid	2.01 ± 0.27	2.01 ± 0.33	0.99	2.67 ± 0.58	1.86 ± 0.25	0.14
Valeric acid	1.64 ± 0.36	1.38 ± 0.28	0.34	1.51 ± 0.35	2.80 ± 0.74	0.13
Hexanoic acid	0.30 ± 0.14	0.27 ± 0.08	0.78	0.31 ± 0.13	0.20 ± 0.08	0.38

Values are expressed as the mean ± standard error (SE), *n* = 14–16. * A linear mixed-effects model was used to test statistical significance.

## Supplementary Materials

The following are available online at https://www.mdpi.com/2072-6643/12/11/3408/s1, Figure S1: CONSORT trial flow diagram.
