# Supplementary material for: The Effects of Non-Nutritive Artificial Sweeteners, Aspartame and Sucralose, on the Gut Microbiome in Healthy Adults: Secondary Outcomes of a Randomized Double-Blinded Crossover Clinical Trial
Source: Nutrients. 2020 Nov 6;12(11):3408. doi: 10.3390/nu12113408 (PMC7694690; doi:10.3390/nu12113408)
Supplement: Supplementary file 1 [file nutrients-12-03408-s001.pdf]

Figures

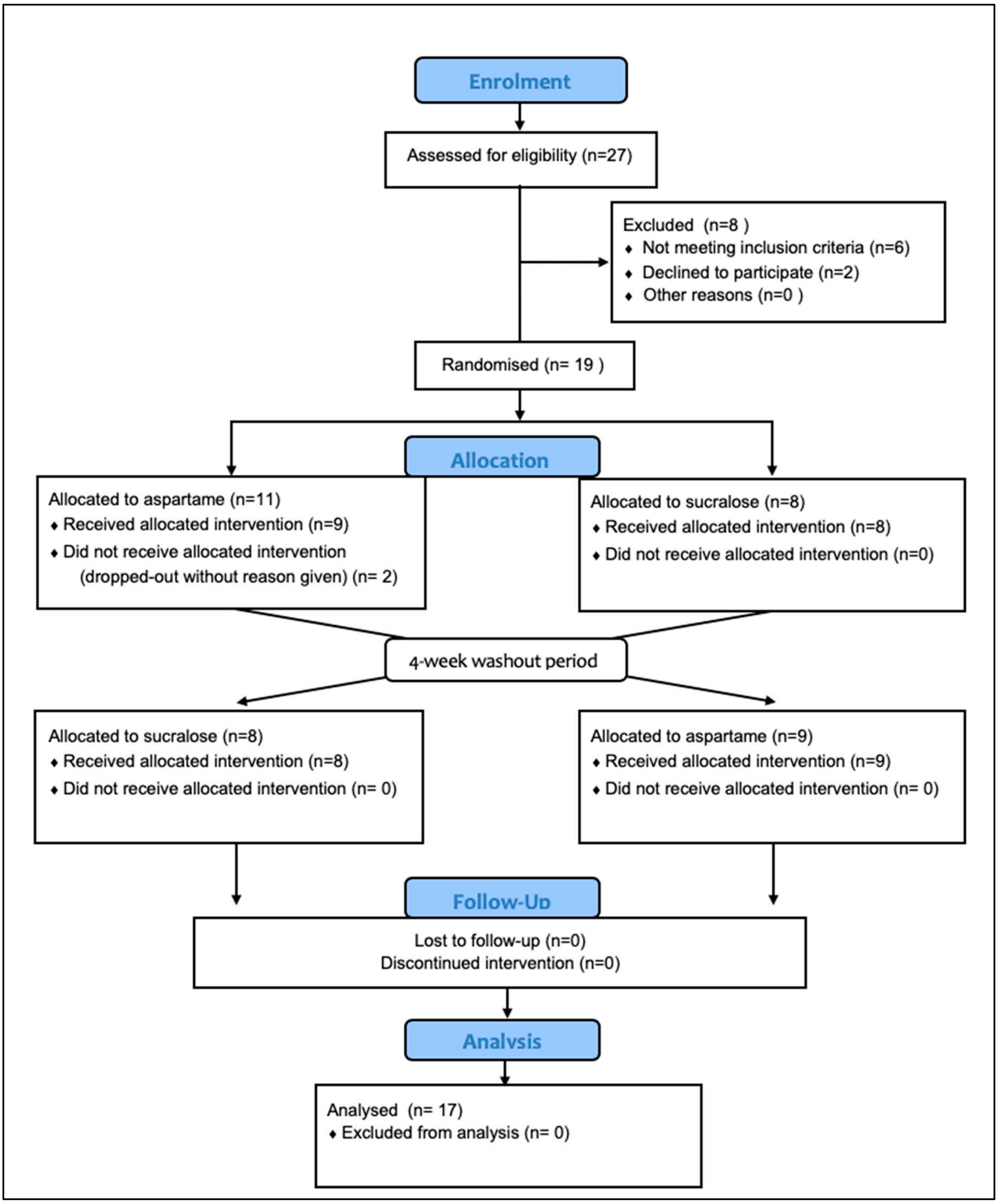

Figure S1. CONSORT trial flow diagram. CONSORT, Consolidated Standards of Reporting Trials [Ahmad, S.Y.; Friel, J.K.; MacKay, D.S. The effect of the artificial sweeteners on glucose metabolism in healthy adults: A randomized, double-blinded, crossover clinical trial. Appl. Physiol. Nutr. Metab. 2020, 45, 606–612].
